# Supplementary material for: Artificial intelligence exceeds humans in epidemiological job coding
Source: Commun Med (Lond). 2023 Nov 4;3:160. doi: 10.1038/s43856-023-00397-4 (PMC10625577; doi:10.1038/s43856-023-00397-4)
Supplement: Supplementary file 1 — Reporting Summary [file 43856_2023_397_MOESM1_ESM.pdf]

nature portfolio

Corresponding author(s): E.L. van den Broek
M.A. Langezaal

Last updated by author(s): Oct 25, 2023

Reporting Summary

Nature Portfolio wishes to improve the reproducibility of the work that we publish. This form provides structure for consistency and transparency in reporting. For further information on Nature Portfolio policies, see our Editorial Policies and the Editorial Policy Checklist.

Statistics

For all statistical analyses, confirm that the following items are present in the figure legend, table legend, main text, or Methods section.

n/a	Confirmed	
☐	☒	The exact sample size (*n*) for each experimental group/condition, given as a discrete number and unit of measurement
☐	☒	A statement on whether measurements were taken from distinct samples or whether the same sample was measured repeatedly
☐	☒	The statistical test(s) used AND whether they are one- or two-sided<br>*Only common tests should be described solely by name; describe more complex techniques in the Methods section.*
☒	☐	A description of all covariates tested
☐	☒	A description of any assumptions or corrections, such as tests of normality and adjustment for multiple comparisons
☐	☒	A full description of the statistical parameters including central tendency (e.g. means) or other basic estimates (e.g. regression coefficient) AND variation (e.g. standard deviation) or associated estimates of uncertainty (e.g. confidence intervals)
☐	☒	For null hypothesis testing, the test statistic (e.g. *F*, *t*, *r*) with confidence intervals, effect sizes, degrees of freedom and *P* value noted<br>*Give P values as exact values whenever suitable.*
☒	☐	For Bayesian analysis, information on the choice of priors and Markov chain Monte Carlo settings
☒	☐	For hierarchical and complex designs, identification of the appropriate level for tests and full reporting of outcomes
☐	☒	Estimates of effect sizes (e.g. Cohen's *d*, Pearson's *r*), indicating how they were calculated

Our web collection on statistics for biologists contains articles on many of the points above.

Software and code

Policy information about availability of computer code

Data collection

The Constances, Asialymph, and Lifework datasets were obtained from the authors of the original articles of these datasets.

For preprocessing of the data and the development of the classification models the following Python packages were used:
flair 0.8.0.post1
nltk 3.6.2
numpy 1.19.5
pandas 1.2.4
scikit-learn 0.24.1
tokenizers 0.10.2
xgboost 1.5.2

The custom code of the preprocessing and development pipeline will be available on Zenodo.

April 2023

Data analysis

For classification model evaluation, the following Python packages were used:
numpy 1.19.5
pandas 1.2.4
scikit-learn 0.24.1
xgboost 1.5.2

For the statistical analysis, SPSS 28.0.1.0 was used.

For manuscripts utilizing custom algorithms or software that are central to the research but not yet described in published literature, software must be made available to editors and reviewers. We strongly encourage code deposition in a community repository (e.g. GitHub). See the Nature Portfolio guidelines for submitting code & software for further information.

Data

Policy information about availability of data

All manuscripts must include a data availability statement. This statement should provide the following information, where applicable:

- Accession codes, unique identifiers, or web links for publicly available datasets
- A description of any restrictions on data availability
- For clinical datasets or third party data, please ensure that the statement adheres to our policy

The Constances [24], Asialymph [25], and Lifework [26] datasets are not publicly available. Access to these datasets should be requested from the authors of the original studies. The Formaldehyde [43] and Silica [44] JEMs can be consulted on the Exp-pro portal of ``Santé publique France'' at https://exppro.santepubliquefrance.fr/matgene. Access to the computer files of the matrices for their use in the context of epidemiological studies should be requested from the authors of the original study. The ALOHA [45] and DOM [46] JEMs are not publicly available and should be requested from the authors of the original study. Source data underlying the graphs in Figure 2 are available as Supplementary Data 1.

Research involving human participants, their data, or biological material

Policy information about studies with human participants or human data. See also policy information about sex, gender (identity/presentation), and sexual orientation and race, ethnicity and racism.

Reporting on sex and gender	In the current study, no information or data on sex and/or gender has been used. For information on the reporting on sex and gender in the Constances, Asialymph, and/or Lifework datasets, we refer to the original articles of those databases.
Reporting on race, ethnicity, or other socially relevant groupings	In the current study, no information or data has been used on race, ethnicity, or other socially relevant groupings. For information on the reporting on race, ethnicity, or other socially relevant groupings in the Constances, Asialymph, and/or Lifework datasets, we refer to the original articles of those databases.
Population characteristics	For descriptive statistics of the job description and corresponding occupational code data we refer to Table 2 and 3 in the main article. For information on all population characteristics of the Constances, Asialymph, and/or Lifework datasets, we refer to the original articles of those databases.
Recruitment	For information on the recruiting process of the Constances, Asialymph, and/or Lifework datasets, we refer to the original articles of those databases.
Ethics oversight	For information on possible ethics oversights in the Constances, Asialymph, and/or Lifework datasets, we refer to the original articles of those databases.

Note that full information on the approval of the study protocol must also be provided in the manuscript.

Field-specific reporting

Please select the one below that is the best fit for your research. If you are not sure, read the appropriate sections before making your selection.

☒ Life sciences ☐ Behavioural & social sciences ☐ Ecological, evolutionary & environmental sciences

For a reference copy of the document with all sections, see nature.com/documents/nr-reporting-summary-flat.pdf

Life sciences study design

All studies must disclose on these points even when the disclosure is negative.

Sample size	For the development of the classification models we used 637,148, 36,179, and 12,120 manually coded job episodes from the Constances, Asialymph, and Lifework datasets, respectively.
Data exclusions	Entries with incomplete codes or missing/empty fields were excluded.
Replication	For the development and evaluation of all classification models, replication was successful. This was done by developing separate Python scripts for development and evaluation, in which the random values were set to those used in the original development and evaluation of the classification models.

Randomization	During the development of the classification models, all datasets were randomly divided using a split of 60% for training, 30% for testing, and 10% for validation. This randomization was realized through the usage of the "train_test_split" function of the scikit-learn Python package.
Blinding	The random distribution of entries into the train, test, and validation subsets of all datasets was done automatically by the mentioned Python package, leaving the researchers without direct control or knowledge of this allocation process.

Reporting for specific materials, systems and methods

We require information from authors about some types of materials, experimental systems and methods used in many studies. Here, indicate whether each material, system or method listed is relevant to your study. If you are not sure if a list item applies to your research, read the appropriate section before selecting a response.

Materials & experimental systems

n/a	Involved in the study
☒	☐ Antibodies
☒	☐ Eukaryotic cell lines
☒	☐ Palaeontology and archaeology
☒	☐ Animals and other organisms
☒	☐ Clinical data
☒	☐ Dual use research of concern
☒	☐ Plants

Methods

n/a	Involved in the study
☒	☐ ChIP-seq
☒	☐ Flow cytometry
☒	☐ MRI-based neuroimaging
