# Supplementary material for: Artificial intelligence exceeds humans in epidemiological job coding
Source: Commun Med (Lond). 2023 Nov 4;3:160. doi: 10.1038/s43856-023-00397-4 (PMC10625577; doi:10.1038/s43856-023-00397-4)
Supplement: Supplementary file 2 — Supplementary Information [file 43856_2023_397_MOESM2_ESM.pdf]

Artificial intelligence exceeds humans in epidemiological job coding

Mathijs A. Langezaal[1,2*], Egon L. van den Broek[2*], Susan Peters[3], Marcel Goldberg[1], Grégoire Rey[4], Melissa C. Friesen[5], Sarah J. Locke[5], Nathaniel Rothman[5], Qing Lan[5] and Roel C. H. Vermeulen[3]

[1*]Population-Based Epidemiological Cohorts Unit UMS11, INSERM, 16 Avenue Paul Vaillant Couturier, Paris, 94807, Villejuif, France.

[2*]Department of Information and Computing Sciences, Utrecht University, Princetonplein 5, Utrecht, 3584CC, Utrecht, The Netherlands.

[3]Institute for Risk Assessment Sciences, Utrecht University, Yalelaan 1, Utrecht, 3584CL, Utrecht, The Netherlands.

[4]Center for Epidemiology on Medical Causes of Death (CépiDc), INSERM, Le Kremlin-Bicêtre, France.

[5]Occupational and Environmental Epidemiology Branch, Division of Cancer Epidemiology and Genetics, National Cancer Institute, Bethesda, Maryland, USA.

*Corresponding author(s). E-mail(s): m.a.langezaal@uu.nl; vandenbroek@acm.org;

Supplementary Methods

Manual coding procedures

The original article of the Lifework [1] data set did not detail the manual coding procedures. However, the corresponding author of the article has now provided the manual coding procedures, as described in the main article.

Description of XGBoost

XGBoost [2] computes a series of individually weak Classification and Regression Trees (CARTs) and combines them to create one, better-performing ensemble. For a given dataset $\mathcal{D} = \{(x_i, y_i)\}$ with n examples and m features, a prediction ($\hat{y_i}$) is assigned to an instance by summing the scores (i.e., the leaf weights) of the leaves of each CART in the ensemble for that instance. This is defined as:

$$\hat{y}_i = \sum_{k=1}^{K} f_k(x_i), f_k \in \mathcal{F}, \tag{1}$$

where K is the number of CARTs, and f is a CART out of the set of all possible CARTs $\mathcal{F}$. In the current context of multiclass classification with L outcome categories, XGBoost computes L CARTs with a binary outcome in each iteration. Consequently, the algorithm computes an ensemble of L binary outcome ensembles, each containing K CARTs to classify job descriptions x_i into an occupational code y_i.

To learn the set of CARTs used in the ensemble, the following regularized objective is minimized:

$$\text{obj}^{(t)} = \sum_{i=1}^{n} l(y_i, \hat{y}_i^{(t)}) + \sum_{k=1}^{t} \Omega(f_k), \tag{2}$$

where l is the loss function that measures the residual error between the target y_i and prediction $\hat{y_i}$. To reduce the chance of overfitting, the second term $\Omega(f_i)$ penalizes the complexity of the model [2]. This is the sum of the complexity of each individual CART in the ensemble. To define the complexity of a CART $\Omega(f)$, the definition of a CART $f(x)$ is first defined as:

$$w \in R^T, q : R^d \to \{1, 2, \cdots, T\}, f_t(x) = w_{q(x)}. \tag{3}$$

Here, w is the vector of scores on the leaves of CART structure q, and T is the number of leaves. Using this definition, XGBoost defines the complexity of a CART $\Omega(f)$ as:

$$\gamma T + \frac{1}{2}\lambda \sum_{j=1}^{T} w_j^2, \tag{4}$$

where w_j is the score on the j-th leaf of a CART, and γ and λ are optimizable hyperparameters controlling the penalty for the number of leaves and the magnitude of the leaf weights, respectively.

Each ensemble is computed in an additive manner, where a new CART is fitted to the residual errors of the previous iteration. Using Eq. (2), the objective function at the t-th step becomes:

$$\mathrm{obj}^{(t)} = \sum_{i=1}^{n} l(y_i, \hat{y}_i^{(t-1)} + f_t(x_i)) + \Omega(f_t). \tag{5}$$

When a new CART is trained, the hyperparameters *sumbsample*, *colsample_ bytree*, and *colsample_ bylevel* specify what proportion of the training data is used. Here, *subsample* is the fraction of the training samples used in each boosting round. Lowering this value could increase computational speed and reduce the chance of overfitting [2]. Hyperparameters *colsample_ bytree* and *colsample_ bylevel* are the subsample ratios of columns that are used during training of each CART and level, respectively. This is a method used in RandomForest algorithms, and could prevent overfitting [3]. However, because the subsampling of columns could result in the loss of important interactions between features of the job descriptions, they were set to include all columns [4].

Depending on the problem the Machine Learning (ML) model is solving (e.g., regression or classification problems), different loss functions might be required. Because of the difference in computational complexity of loss functions, XGBoost uses the Taylor expansion up to the second order to approximate the value of the loss function around a given point. For example, this could be used in the current context to approximate the local minimum of a function using logistic loss as its loss function. Applying Taylors expansion up to the second order to Eq. (5) results in the following objective function:

$$\mathrm{obj}^{(t)} \approx \sum_{i=1}^{n} [g_i f_t(x_i) + \frac{1}{2} h_i f_t^2(x_i)] + \Omega(f_t), \tag{6}$$

where

$$\begin{aligned} g_i &= \partial_{\hat{y}_i^{(t-1)}} l(y_i, \hat{y}_i^{(t-1)}), \\ h_i &= \partial^2_{\hat{y}_i^{(t-1)}} l(y_i, \hat{y}_i^{(t-1)}). \end{aligned} \tag{7}$$

As the instance set of leaf j is defined as $I_j = \{i|q(x_i) = j\}$, Eq. (6) is rewritten by expanding Ω as:

$$\begin{aligned}\text{obj}^{(t)} &\approx \sum_{i=1}^{n}[g_i w_{q(x_i)} + \frac{1}{2}h_i w_{q(x_i)}^2] + \gamma T + \frac{1}{2}\lambda\sum_{j=1}^{T} w_j^2 \\ &= \sum_{j=1}^{T}[(\sum_{i\in I_j} g_i)w_j + \frac{1}{2}(\sum_{i\in I_j} h_i + \lambda)w_j^2] + \gamma T.\end{aligned} \tag{8}$$

Consequently, the optimal leaf weight w_j^* of leaf j for a fixed CART structure $q(x)$ can be computed by:

$$-\frac{\sum_{i\in I_j} g_i}{\sum_{i\in I_j} h_i + \lambda}. \tag{9}$$

To secure possible overfitting, the weights of the new features are reduced by hyperparameter λ and restricted to a maximum weight by *max_delta_step* with each boosting step. As λ has a direct influence on h_i, increasing its value will reduce the chance of weights getting too large and thus reduce the chance of overfitting. As seen in Eq. (9), h_i can become very small with imbalanced datasets such as the current ones (see Table 2), resulting in very large weights. Restricting the maximum weight will ensure that one leaf does not have too much influence on $\hat{y}$, making the model more conservative. To further prevent overfitting, the XGBoost algorithm applies shrinkage of leaf weights as introduced by Friedman [5]. Shrinkage scales newly added weights w_j^* by a factor η to reduce the influence of an individual CART. This leaves space for future CARTs to improve the ensemble.

To calculate the optimal corresponding leaf weight w_j^*, the following equation is used:

$$\text{obj}^* = -\frac{1}{2}\sum_{j=1}^{T}\frac{(\sum_{i\in I_j} g_i)^2}{\sum_{i\in I_j} h_i + \lambda} + \gamma T. \tag{10}$$

This equation is also used as a scoring function to measure the quality of a CART structure $q(x)$.

Because of the amount of different possible combinations of splits, enumerating all possible CART structures $q(x)$ is intractable. Hence, a greedy algorithm uses the following formula to iteratively find the split providing the most information gain.

$$Gain = \frac{1}{2}\left[\frac{(\sum_{i\in I_L} g_i)^2}{\sum_{i\in I_L} h_i + \lambda} + \frac{(\sum_{i\in I_R} g_i)^2}{\sum_{i\in I_R} h_i + \lambda} - \frac{(\sum_{i\in I} g_i)^2}{\sum_{i\in I} h_i + \lambda}\right] - \gamma. \tag{11}$$

Here, I_L and I_R are the instance sets of the left and right node after the split, respectively. The algorithm will compute the next best splits iteratively until no further gain can be found (i.e., gain <0) or the CART has reached

its maximum depth. Both stopping conditions can be empirically optimized through the hyperparameters γ and *max_ depth*, respectively. Here, γ is the minimum gain each split should produce. In the current domain, too large values of γ could result in the addition of splits including irrelevant interactions between features of job descriptions [4]. Whereas a too small value will result in important interactions being missed. The hyperparameter *max_ depth* refers to the maximum depth of a CART. By lowering this value the CARTs in the model will contain fewer splits, making it less likely to overfit on the training data [6].

Supplementary Tables

Table S1: Exposure assessment accuracy (Acc., %) and κ of the classification models using the Formaldehyde-JEM, Silica-JEM, ALOHA-JEM and DOM-JEM. Accuracy and κ are given for two groups 1) All individuals (PCS&NAF: N = 22913, ISCO-88: N = 10,689, ISCO-68: N = 3,569), and 2) Exposed individuals. For the second group, the number of exposed individuals (N) is given for each exposure.

	All Individuals		Exposed Individuals		
Occupational exposure	*Acc.*	κ	N	*Acc.*	κ
Formaldehyde-JEM (PCS&NAF)	*98.09*	*0.84*	*1444*	*81.85*	*0.81*
Silica-JEM (PCS&NAF)	*98.41*	*0.67*	*592*	*61.82*	*0.61*
ALOHA-JEM (ISCO-88)	*75.05*	*0.70*	*6755*	*65.58*	*0.63*
Biological dust	87.86	0.75	3624	78.52	0.45
Mineral dust	85.64	0.75	4428	78.27	0.62
Gasfumes	83.69	0.73	5900	78.68	0.47
VGDF	84.06	0.76	6663	79.80	0.63
All pesticides	95.25	0.83	1571	84.23	0.62
Herbicides	98.03	0.91	1272	87.53	0.54
Insecticides	97.40	0.89	1371	87.69	0.56
Fungicides	95.94	0.84	1460	84.72	0.63
Aromatic solvents	91.38	0.77	2561	79.22	0.34
Chlorinated solvents	92.32	0.63	1181	59.67	0.38
Other solvents	89.75	0.65	1904	66.22	0.31
Metals	91.23	0.61	1271	57.12	0.37
DOM-JEM (ISCO-68)	*84.19*	*0.59*	*884*	*49.77*	*0.47*
Asbestos	95.37	0.55	220	48.64	0.11
Chromium	98.66	0.42	55	30.90	0.16
DME	95.38	0.57	233	50.64	0.18
Nickel	98.90	0.38	44	25.00	0.12
PAH	95.71	0.58	189	55.56	0.04
Silica	98.18	0.46	72	40.28	0.03
Animal	98.37	0.51	71	42.25	0.24
Biological dust	91.85	0.63	484	59.71	0.24
Endotoxin	94.56	0.59	266	56.77	0.12

Table S2: **For each occupational exposure in the ALOHA-JEM and DOM-JEM, exposure assessment accuracy (Acc., %) of respectively the ISCO-88 and ISCO-68 classification models per exposure level (EL).** No, Low, and High respectively represent no exposure, low exposure, and high exposure levels, as indicated by the ALOHA-JEM and DOM-JEM. *N* indicates the count of job episodes for each exposure level.

ALOHA-JEM (ISCO-88)				DOM-JEM (ISCO-68)			
Occupational exposure	EL	*N*	*Acc.*	Occupational exposure	EL	*N*	*Acc.*
Biological dust	No	7065	92.76	Asbestos	No	3349	98.44
	Low	3023	83.59		Low	193	51.29
	High	601	60.89		High	27	29.62
Mineral dust	No	6261	90.95	Chromium	No	3514	99.71
	Low	2282	76.24		Low	35	31.40
	High	2146	82.61		High	20	30.00
Gasfumes	No	4789	89.95	DME	No	3336	98.50
	Low	4663	84.21		Low	204	49.50
	High	1237	61.68		High	29	58.62
VGDF	No	4026	91.20	Nickel	No	3525	99.82
	Low	3448	80.80		Low	23	21.73
	High	3215	80.24		High	21	28.57
All pesticides	No	9118	97.26	PAH	No	3380	97.95
	Low	330	58.78		Low	182	57.14
	High	1241	95.24		High	7	14.28
Herbicides	No	9417	99.55	Silica	No	3497	99.37
	Low	133	60.15		Low	69	42.02
	High	1139	95.61		High	3	0.00
Insecticides	No	9318	98.26	Animal	No	3498	99.51
	Low	141	56.02		Low	40	52.50
	High	1230	95.85		High	31	29.03
Fungicides	No	9229	97.82	Biological dust	No	3085	96.88
	Low	307	63.84		Low	404	63.61
	High	1153	94.88		High	80	40.00
Aromatic solvents	No	8128	95.34	Endotoxin	No	3303	97.60
	Low	2380	81.59		Low	241	59.75
	High	181	75.58		High	25	28.00
Chlorinated solvents	No	9508	96.68				
	Low	844	62.08				
	High	337	62.61				
Other solvents	No	8785	95.09				
	Low	1715	67.05				
	High	189	77.77				
Metals	No	9418	96.16				
	Low	802	56.10				
	High	469	64.91				

Table S3: **Accuracy (*Acc.*, %) per major group of the NAF classification model on the highest coding level.**

Major group	% in group	*Acc.*
A: Agriculture, sylviculture et pêche	0.85	71.88
B: Industries extractives	0.18	52.17
C: Industrie manufacturière	11.72	75.60
D: Production et distribution d'électricité, de gaz, de vapeur et d'air conditionné	2.79	94.24
E: Production et distribution d'eau ; assainissement, gestion des déchets et dépollution	0.41	73.83
F: Construction	2.14	74.81
G: Commerce ; réparation d'automobiles et de motocycles	11.51	84.49
H: Transports et entreposage	3.53	86.36
I: Hébergement et restauration	4.55	92.16
J: Information et communication	1.92	85.51
K: Activités financières et d'assurance	5.37	95.91
L: Activités immobilières	0.59	79.60
M: Activités spécialisées, scientifiques et techniques	5.83	84.86
N: Activités de services administratifs et de soutien	3.34	79.13
O: Administration publique	11.27	89.03
P: Enseignement	11.29	93.91
Q: Santé humaine et action sociale	16.12	94.47
R: Arts, spectacles et activités récréatives	2.85	79.56
S: Autres activités de services	2.94	78.66
T: Activités des ménages en tant qu'employeurs	0.56	67.36
U: Activités extra-territoriales	0.12	61.29

Table S4: **Accuracy (*Acc.*, %) per major occupational group of the PCS classification model on the highest coding level.**

Major occupational group	% in group	*Acc.*
1: Agriculteurs exploitants	0.09	46.51
2: Artisans, commerçants et chefs d'entreprise	1.68	43.39
3: Cadres et professions intellectuelles supérieures	19.48	80.77
4: Professions Intermédiaires	31.49	81.97
5: Employés	27.29	90.57
6: Ouvriers	16.54	88.00
7: Retraités	<0.01	33.33
8: Autres personnes sans activité professionnelle	0.31	32.45

Table S5: **Accuracy (*Acc.*, %) per major occupational group of the ISCO-88 classification model on the highest coding level.**

Major occupational group	% in group	*Acc.*
1: Legislators, senior officials and managers	6.94	66.04
2: Professionals	7.12	78.49
3: Technicians and associate professionals	14.54	70.96
4: Clerks	9.11	74.34
5: Service workers and shop and market sales workers	13.03	86.55
6: Skilled agricultural and fishery workers	12.90	96.12
7: Craft and related trades workers	12.24	67.17
8: Plant and machine operators and assemblers	16.73	75.60
9: Elementary occupations	7.40	63.53
0: Armed Forces	0.00	-

Table S6: Accuracy (*Acc.*, %) per major occupational group of the ISCO-68 classification model on the highest coding level.

Major occupational group	% in group	*Acc.*
0/1: Professional, Technical and Related Workers	18.10	70.38
2: Administrative and Managerial Workers	14.10	78.64
3: Clerical and Related Workers	29.28	80.68
4: Sales Workers	9.17	68.59
5: Service Workers	11.42	75.36
6: Agricultural, Animal Husbandry and Forestry Workers, Fishermen and Hunters	2.25	38.23
7/8/9: Production and Related Workers, Transport Equipment Operators and Labourers	15.68	53.59

Supplementary References

[1] Reedijk, M. *et al.* Cohort profile: LIFEWORK, a prospective cohort study on occupational and environmental risk factors and health in the Netherlands. *BMJ Open* **8**, e018504 (2018) .

[2] Chen, T. & Guestrin, C. XGBoost: a scalable tree boosting system. In *Proceedings of the 22nd ACM SIGKDD International Conference on Knowledge Discovery and Data Mining* 785–794 (ACM, 2016).

[3] Breiman, L. Random Forests. *Machine Learning* **45**, 5–32 (2001) .

[4] Schierholz, M. & Schonlau, M. Machine learning for occupation coding—a comparison study. *Journal of Survey Statistics and Methodology* **9**, 1013–1034 (2021) .

[5] Friedman, J. H. Stochastic gradient boosting. *Computational Statistics & Data Analysis* **38**, 367–378 (2002) .

[6] Bertsimas, D. & Dunn, J. Optimal classification trees. *Machine Learning* **106**, 1039–1082 (2017) .
