# Supplementary material for: Artificial intelligence exceeds humans in epidemiological job coding
Source: Commun Med (Lond). 2023 Nov 4;3:160. doi: 10.1038/s43856-023-00397-4 (PMC10625577; doi:10.1038/s43856-023-00397-4)
Supplement: Supplementary file 4 — Description of Additional Supplementary Files [file 43856_2023_397_MOESM4_ESM.pdf]

Description of Additional Supplementary Files

File Name: Supplementary Data 1
Description: Underlying data of Figure 2
